# Asking sensitive questions in conservation using Randomised Response Techniques

**DOI:** 10.1016/j.biocon.2021.109191

**Published:** 2021-08

**Authors:** Harriet Ibbett, Julia P.G. Jones, Freya A.V. St John

**Affiliations:** School of Natural Sciences, College of Environmental Sciences & Engineering, Bangor University, Thoday Building, Deiniol Road, Bangor, Gwynedd LL57 2DF, UK

**Keywords:** Specialised questioning techniques, Indirect questioning, Non-compliance, Rule-breaking, Natural resource management, Sensitivity bias

## Abstract

Conservation increasingly seeks knowledge of human behaviour. However, securing reliable data can be challenging, particularly if the behaviour is illegal or otherwise sensitive. Specialised questioning methods such as Randomised Response Techniques (RRTs) are increasingly used in conservation to provide greater anonymity, increase response rates, and reduce bias. A rich RRT literature exists, but successfully navigating it can be challenging. To help conservationists access this literature, we summarise the various RRT designs available and conduct a systematic review of empirical applications of RRTs within (*n* = 32), and beyond conservation (*n* = 66). Our results show increased application of RRTs in conservation since 2000. We compare the performance of RRTs against known prevalence of the sensitive behaviour and relative to other questioning techniques to assess how successful RRTs are at reducing bias (indicated by securing higher estimates). Findings suggest that RRT applications in conservation were less likely than those in other disciplines to provide prevalence estimates equal to, or higher than those derived from direct questions. Across all disciplines, we found reports of non-compliance with RRT instructions were common, but rarely accounted for in study design or analysis. For the first time, we provide conservationists considering RRTs with evidence on what works, and provide guidance on how to develop robust designs suitable for conservation research contexts. We highlight when alternate methods should be used, how to increase design efficiency and improve compliance with RRT instructions. We conclude RRTs are a useful tool, but their performance depends on careful design and implementation.

## Highlights

• Randomised Response Techniques (RRTs) are increasingly used in conservation.
• Evidence from a systematic review shows RRT performance in conservation varies.
• Non-compliance with RRT instructions is common, but rarely accounted for.
• RRTs are not appropriate for all research questions.
• To be successful, RRTs require careful design and implementation.

## 1 Introduction

Conservationists increasingly seek reliable information about people's behaviour, including illegal or otherwise sensitive topics where people may not be comfortable answering truthfully (Solomon et al., 2007; Cinner, 2018). Securing reliable estimates about the proportion of the population engaged in rule-breaking, as well as what drives non-compliance, is critical for the development of effective conservation interventions (St John et al., 2013). It is well understood across a range of social research disciplines, particularly when the topic of investigation is sensitive, that respondents may adjust their answers to appear more socially acceptable (social desirability bias), or refuse to answer altogether (non-response bias, Krumpal, 2013; Tourangeau and Yan, 2007). Specialised questioning techniques such as the Unmatched Count Technique (UCT) (Droitcour et al., 1991) and Randomised Response Techniques (RRTs) (Warner, 1965) have been developed to overcome these biases. These methods provide respondents with greater anonymity when answering sensitive questions (Chaudhuri and Christofides, 2013) and are grounded in the premise that respondents are more likely to answer truthfully when question design protects them from revealing incriminating information (Warner, 1965). Within conservation, there is growing interest in using specialised questioning techniques to derive more reliable estimates when researching potentially sensitive behaviours (Arias et al., 2020; Cerri et al., 2021; Hinsley et al., 2018), but to be effective, these techniques require robust design underpinned by good understanding of their advantages and limitations (Hinsley et al., 2018; Nuno and St John, 2015). Here, we describe the various RRT designs, conduct a systematic review of their application, and provide evidence on what works. In doing so, we aim to improve conservationists' understanding of the design considerations, alongside potential pitfalls.

**Fig. 1:**
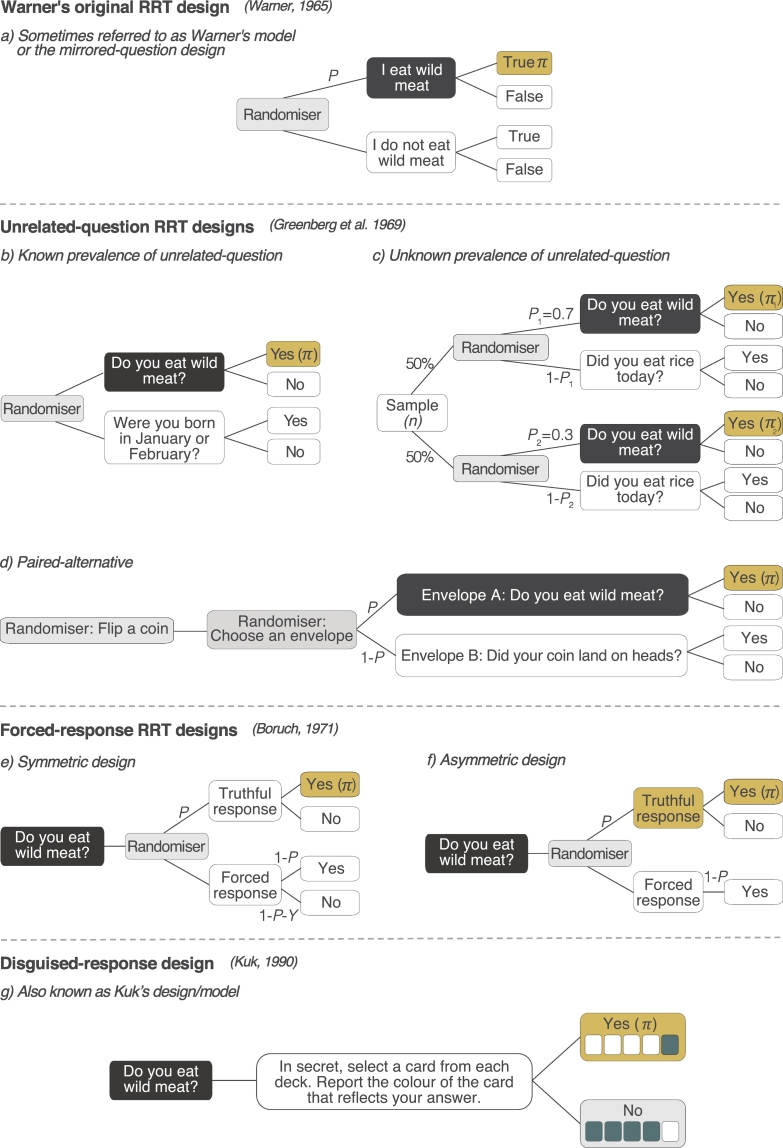
Probability trees showing various RRT designs used to estimate the proportion of a population engaged in a sensitive behaviour, such as consuming wildmeat. Light grey boxes indicate the point at which a randomising device is used; dark grey boxes indicate the sensitive question; yellow boxes and *π* indicate the prevalence estimate. *P* = probability of answering a question truthfully (forced-response designs), or being asked to answer the sensitive question (unrelated-question designs), *Y* = probability of providing a forced-yes response. (For interpretation of the references to colour in this figure legend, the reader is referred to the web version of this article.)

Developed by Warner in 1965 to overcome bias, RRTs work by enabling interviewees to respond with answers that provide information on a probability basis (Warner, 1965). In Warner's original RRT design (sometimes referred to as Warner's model, or the mirrored-question design, Blair et al., 2015), respondents are presented with a randomising device (e.g., a spinner), which they use to randomly select a statement relating to a sensitive topic. Respondents are asked to report if the statement selected by the randomiser is true or false for them (Fig. 1a). The sample-level prevalence of the sensitive behaviour is calculated using the known probability of answering the sensitive statement (*ρ*), the total number of ‘yes’ responses (Υ), and the total sample size (n) (Box 1). By protecting respondents (who never reveal which statement they answered), and enumerators (who cannot tell which statement was answered), RRTs can reduce bias and yield higher estimates than asking people sensitive questions directly (hereafter, direct questions) (Dietz et al., 2013; Lensvelt-Mulders et al., 2005a). Consequently, RRTs have been applied extensively to investigate sensitive topics including drug-use, sexual behaviour and abortion (de Jong et al., 2012; Lara et al., 2006; Stubbe et al., 2014).

Box 1 **How to calculate estimates of prevalence using RRT (Fox, 2016; Warner, 1965):**

$$\pi =\frac{\frac{\Upsilon }{n}\;+\;\rho -1}{2\rho -1}$$

Variance, which considers the additional uncertainty added by the randomisation process, is calculated as:

$\mathrm{Variance}=\frac{\pi \;\left(1-\pi \right)}{n}+\frac{\rho \left(\;1-\rho \right)}{n\;{\left(2\rho -1\right)}^{2}}$

Alt-text: Box 1

After Warners' inception of the first RRT, it was rapidly recognised that the additional anonymity afforded by the randomisation process came at a cost of efficiency, with estimates associated with high levels of error (Greenberg et al., 1969). As a result, Warners' original design was extensively refined (Blair et al., 2015) and a suite of different RRT designs (also referred to as models) are now available; each optimised to improve administration, reduce error and increase efficiency (Chaudhuri and Mukherjee, 1987; Fox, 2016). Today, a rich literature documenting advances in RRTs and reviewing their efficacy exists (Lensvelt-Mulders et al., 2005b; Umesh and Peterson, 1991). However, successfully navigating it can present challenge to conservationists; there are inconsistencies in nomenclature (e.g. Warner's design and the mirrored-question design are the same) and accessing research requires extensive review of literature across multiple fields. Moreover, many RRT designs were developed and applied in western-educated contexts, yet substantial conservation research occurs in places where literacy and access to education are more limited (Brittain et al., 2020).

Determining which RRT to use is challenging without empirical information about what works. To improve understanding and to guide conservationists, we summarise the various RRT designs and then undertake a systematic review of peer-reviewed literature describing the use of RRTs in conservation. We review the conservation topics studied, countries where it has been used, and the designs applied. Using the wider literature, we assess the performance of RRTs by exploring studies that validated RRT estimates using data on known prevalence, alongside studies that compared RRT estimates to those derived from alternate questioning methods; we then explore which design considerations affect performance. Using findings from our review, along with our own experience, we provide best practice guidelines to conservationists deciding whether, and how, to use RRTs.

### 1.1 The unrelated-question, and paired alternative RRT designs

One of the most used post-Warner designs is the unrelated-question RRT. First proposed by Simmons et al. (1967) and improved by Greenberg et al. (1969), instead of randomly selecting from two statements about the *same* topic, respondents randomly select a question from *two different* topics (Horvitz et al., 1976). One question is innocuous and completely unrelated to the sensitive topic, the other is the sensitive question of interest. A randomising device is used to determine *which* question is answered, while the possible responses to both questions remain the same (e.g., yes, or no). In Idaho, USA, Schill and Kline (1995) successfully used an unrelated-question design to estimate non-compliance of anglers with fishing regulations.

The unrelated-question design is improved further by asking an unrelated-question for which probability of an affirmative (yes) response is *known* (Fig. 1b), for example, asking about a respondent's birth month, population-level data on which can be obtained from census records (Boruch, 1971). Even if the level of the unrelated question is *unknown*, prevalence of the sensitive characteristic can still be obtained (albeit with lower statistical efficiency) by splitting the sample into two and assigning each a different probability of answering the sensitive question (e.g. sample 1 has 0.7 chance of answering the sensitive question, while the probability for sample 2 is 0.3) (Fox, 2016) (Fig. 1c). Chu et al. (2018) adopted this approach in an online survey to research misuse of IT software and the internet by employees in the workplace.

Where obtaining data on an unrelated-question is challenging, or it is impractical to split the sample in two, a *paired-alternative* design (also known as the *two unrelated-questions* design (Fox, 2016)) can be used. This design introduces an additional randomisation process, the outcome of which forms the subject of the unrelated-question (Fig. 1d). For example, in their study investigating illegal resource use in Kibale National Park, Uganda, Solomon et al. (2007) first asked respondents to flip a coin, and then presented two identical envelopes and asked respondents to select one. Inside, one envelope contained a card featuring an image of the ‘head’ side of a coin, the other included a photograph depicting an illegal activity (e.g. setting snares inside the park). When respondents looked at the card in the envelope, they were asked to say “yes”, if the card showed the head of a coin and they had flipped a head, or “no” if the card showed the head of a coin, and they had not flipped a head. If the card in the envelope featured the photograph depicting setting snares, they were asked to honestly report whether they had done the activity. This method can increase efficiency in contexts where questions with known probabilities (e.g., birth months) are poorly known.

### 1.2 The forced-response RRT design

To further improve statistical efficiency and to enhance RRT simplicity, Boruch (1971) developed the forced-response RRT design (also referred to as the forced-alternative (Fox, 2016). Boruch (1971) aimed to eliminate the need for a second topic of enquiry whilst maintaining the randomisation process. The forced-response design uses randomisation to establish *how* a respondent should answer the sensitive question; truthfully (with probability *ρ*), or with a ‘forced’ response (e.g., yes, or no). Within conservation, this design has been applied extensively (e.g. Randriamamonjy et al., 2015; St John et al., 2012; Oyanedel et al., 2017). Two variations of the forced-response exist: the symmetric design, whereby respondents are instructed to provide a truthful answer (e.g., yes, or no), a forced yes or a forced no (Fig. 1e); and the asymmetric design (Fig. 1f), where respondents are instructed to provide either a truthful response (e.g., yes, or no) or one prescribed response, usually “yes”. Although enumerators cannot determine if positive responses are truthful or forced, typically, asymmetric designs assure less protection because enumerators can determine when participants were required to answer the sensitive question (e.g., because people only say no when responding truthfully, Fig. 1f). Even though such a response may not be socially undesirable, it can add discomfort as it decreases anonymity (Fox, 2016).

### 1.3 Kuk's disguised-response RRT design

Despite its efficiency, a key criticism of the forced-response design is that respondents can feel uncomfortable being ‘forced’ to answer yes when their truthful answer would be no (Coutts and Jann, 2011). To overcome this, Kuk (1990) proposed the disguised-response design. Here, respondents are provided two decks of cards, one representing “yes” responses, the other representing “no”. Each deck contains cards of two colours (e.g., blue, and white). In the “yes” deck the ratio of white to blue cards is 4:1, whereas in the “no” deck the ratio is 1:4 (Fig. 1g). To answer a question, respondents secretly select one card from each deck, and report the colour of the card that reflects their answer (i.e. if their answer is yes, they report the colour of the card that they selected from the yes pile) (Blair et al., 2015; Kuk, 1990). Despite its potential, few applications of Kuk's design exist (but see (Van der Heijden et al., 2000), and only one in conservation. Investigating bird hunting in China, Chang et al. (2019) reported no significant difference in estimates between the disguised-response and forced-response designs and found the disguised-response more time consuming as respondents were required to shuffle two decks of cards between questions.

### 1.4 Estimating incidence

RRT designs described so far all capture responses that determine *whether* respondents do something (e.g., eat wild meat), not *how often* they do it. However, RRT designs for estimating incidence do exist (Fox, 2016). Simple adaptions can be made to designs already discussed. For example, the forced-response RRT can be altered so that polychotomous responses are provided (e.g. daily, weekly, monthly, annually, never) instead of dichotomous responses (i.e. ‘yes’ or ‘no’), (de Jong et al., 2012). Asking respondents to provide truthful, or ‘forced’ answers from a wider range of options, each with a known probability can help reduce non-response bias by enabling respondents to provide answers which are more reflective of their true behaviour (Cerri et al., 2018; Cruyff et al., 2007).

**Fig. 2:**
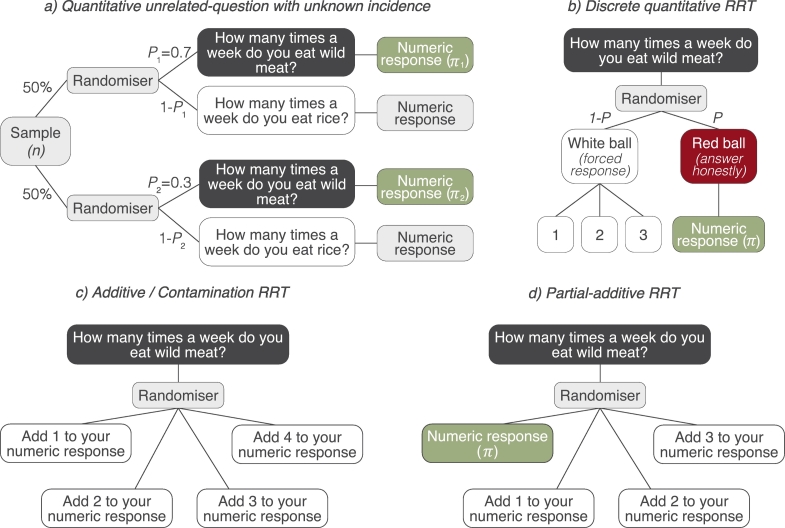
Probability trees for RRT designs that estimate how often (i.e., incidence) sensitive behaviours (such as consuming wild meat) occur. Light grey boxes represent the point at which the randoming device is used; dark grey boxes indicate the sensitive question; green boxes indicate the incidence estimate (*π*). *P* = the probability of answering a question truthfully (forced-response design), or answering the sensitive question (unrelated-question design). (For interpretation of the references to colour in this figure legend, the reader is referred to the web version of this article.)

The RRT can also be used to capture more quantitive estimates of incidence. The *quantitive RRT* design (also known as the *quantitive unrelated-question model*) was first proposed by Greenberg et al. (1971) and works in the same way as the unrelated-question with an unknown prevalence. The sample is split in two, each assigned a different probability of answering the sensitive question, but instead of a binary ‘yes’ or ‘no’ answer, respondents provide a numeric response (Fig. 2a). The mean incidence estimate is calculated using knowledge of the probability of receiving the sensitive question. To further develop the efficiency of this RRT design, Liu and Chow (1976) presented the *discrete-quantitive RRT* (sometimes known as the *quantitive forced-alternative*). This variation builds on the forced-response design and uses a randomiser to determine *how* the respondent should answer. For example, in their study, Liu and Chow (1976) developed a device which contained two different coloured balls (red and white). All the white balls were marked with a number (e.g., 0, 1, 2….) whilst red balls were unmarked. Respondents shook the device, if the ball that appeared in the window was red, they were asked to provide an honest numeric response, if the ball was white, they reported the number on the ball (Fig. 2b). To avoid it being obvious which coloured ball was selected, the numbers listed on white balls all came from a similar distribution to the values expected through honest reporting (i.e., when red balls were selected). Because the probability of reporting white ball numbers is known, efficiency is increased (Fox, 2016). Conteh et al. (2015) adopted this approach to quantify the number of illegal hunting trips undertaken into a forest reserve in Sierra Leone (although note ethical issues with this study see St John et al., 2016).

A further method of note is the *additive* or *contamination* RRT design (Fig. 2c). First proposed by Warner (1971), this design is similar to the *discrete-quantitive RRT* (Liu and Chow, 1976) except all balls are marked with a number from a known distribution and respondents are asked to ‘contaminate’ their response by adding the randomly selected number to their numeric answer (Warner, 1971; Fox, 2016). A variation of this design, known as the *partial-additive RRT* (Gupta and Thornton, 2013) was applied by Robinson et al. (2015) to research reptile pet trade and demand for wildlife. Here, a proportion of respondents were required to answer truthfully (e.g. if they selected a card marked “zero”) and a proportion were asked to add the number on the selected card to their truthful response (Fig. 2d). Kim and Flueck (1978) note that additive models are efficient designs but warned they can increase cognitive load by requiring respondents to sum numeric values.

### 1.5 Are RRTs effective at reducing bias?

Whether RRTs reduce bias is of key interest to conservationists considering their use. One of the key barriers to measuring their performance is the inability to validate results, which requires knowledge about the true prevalence of the sensitive characteristic, ideally at the level of the individual respondent (although often aggregate data are used). A review of 35 years of RRT applications found only six studies where RRT estimates were validated using data on known prevalence (Lensvelt-Mulders et al., 2005b). Of these, a mean discrepancy of 42% was identified between the known prevalence and RRT estimates, with the effect size (i.e., the discrepancy between the values) increasing with question sensitivity. In the absence of reliable data against which to ground-truth estimates, RRT results are often compared to estimates derived from asking people sensitive questions directly; if RRT estimates are significantly higher, then RRT is deemed to have successfully reduced bias (Blair et al., 2015). However, evidence suggests RRTs are not universally successful, with reviews documenting examples where RRT estimates were lower than those of alternate methods (Lensvelt-Mulders et al., 2005b; Umesh and Peterson, 1991).

A range of reasons exist for why RRTs are not always effective. Compared to other specialised questioning techniques, RRTs are reported to be harder for participants to understand (Coutts and Jann, 2011; Davis et al., 2019). Studies have shown that perceptions of privacy can be low (Hoffmann et al., 2017; Höglinger et al., 2016), that randomising devices place excessive cognitive load on respondents (Razafimanahaka et al., 2012; Solomon et al., 2007) and may create distrust towards researchers (Tan et al., 2009) meaning respondents are unwilling or unable to respond to researchers' questions as instructed. Further, although RRTs protect individuals, the wider purpose of the method is to reveal group behaviour. Therefore, where respondents are concerned about incriminating their group (e.g. their community, ethnic group or profession), RRTs may not work (Razafimanahaka et al., 2012)*.*

Moreover, designs such as the forced-response RRT have been shown to evoke psychological resistance where respondents are required to give affirmative answers to actions they did not perform or characteristic they do not possess (Lee & Lee, 2012). Evasive-responding (also called self-protective responding, non-adherence or cheating), occurs when respondents answer “no” regardless of the outcome of the randomising device (John et al., 2018). It may be accidental (i.e. people fail to understand instructions and subsequently answer incorrectly (Clark and Desharnais, 1998)), or deliberate (i.e. individuals anxious to protect themselves and/or avoid being identified as performing a sensitive behaviour purposefully manipulate their responses to avoid sensitive admissions (Moshagen and Musch, 2012)). Measuring the extent to which RRT data suffer from evasive-responses is possible but ethically questionable, as it requires deception. For example, suspecting respondents were failing to follow instructions, Edgell et al. (1982) published one of the first observations of non-adherence. They surreptitiously recorded the outcome of the randomising device and found 25% of respondents reported “no” when instructed to say “yes”.

## 2 Material and methods

### 2.1 Search Criteria & Selection

In March 2019 and April 2020, we conducted systematic searches in Scopus and Web of Science using the search terms “Randomised Response Technique” and “Randomized Response Technique” (English and American spelling). We searched for any peer-reviewed articles published in English language journals, with no constraints on academic discipline, since 1965 (Fig. S1A). The searches provided 1508 articles, including 398 duplicates. The title of each article was scanned to identify whether it mentioned or suggested use of RRTs resulting in 502 articles retained for abstract screening. Abstracts were read to identify a) whether the study collected empirical data using RRTs, and b) whether the study researched a conservation issue including hunting, fishing, wildlife trade or consumption or other forms of natural resource extraction. Conservation articles were included regardless of publication date, while all articles (regardless of discipline) published after 2000 were included, as the last substantial review of RRT was published in 2005 (Lensvelt-Mulders et al., 2005b). In total, 127 articles thought to use RRTs were forwarded for full review. Of these, five were inaccessible. A further 32 were excluded as they either focused on refining RRT design (*n* = 9), did not use RRT (*n* = 15) or discussed RRTs but did not provide prevalence estimates (*n* = 6), one article was not peer reviewed, while one article provided insufficient information. In addition, we identified five conservation articles recently published or published in journals that were not identified in the database searches and added them to the sample.

### 2.2 Data extraction

In total, data were extracted from 98 studies in 95 articles (three articles included two studies) (See Table S1A for a full list of articles reviewed, organised by discipline). For each study, we recorded study location, research topic, and its sensitivity using categories defined by Hinsley et al. (2018) (non-compliant or illegal behaviour (e.g. smuggling or illegal hunting); socially undesirable behaviour (e.g. promiscuity); socially undesirable views (e.g. racism); personal or health (e.g. being HIV positive), and socially desirable behaviours (e.g. recycling). We documented survey administration (sample size, administration mode), RRT method (design used, instructions provided to respondents, randomising device, probability of receiving the sensitive question or providing an honest response, probability of a forced-yes or forced-no response, if pilot study was conducted), and whether RRT estimates were validated using data on known prevalence e.g. government records, or compared to estimates derived using other methods. We recorded the analyses conducted (statistical tests, power analysis, software used), how results were presented, the error reported, and if applicable, whether RRT estimates were statistically higher, lower or the same as those derived using other methods. We documented if authors measured respondents' level of understanding and perceptions of privacy, if free prior informed consent was sought, and whether confidentiality and anonymity was assured. The full review protocol is available in Appendix 1.

### 2.3 Analysis

We present a timeline of key events in the development of RRTs and describe variation in study design, administration, and results. We review performance by summarising results from validation studies, and then assessing whether RRT estimates were significantly higher or lower than estimates derived using other questioning methods. In instances where 95% confidence intervals between estimates overlapped, we concluded there was no significant difference in performance. When RRT estimates were higher than those of other methods, we assumed RRTs were successful at reducing bias, and vice versa when RRT estimates were lower. To investigate which aspects of RRT design affected performance, we ran an ordered logistic regression with a random effect for study using the ‘clmm’ function in the ‘ordinal’ package (Christensen, 2019) in R (v. 3.6.2) (R Core Team, 2019). Due to limited sample sizes, we only used data from studies that used a forced-response or unrelated-question design and compared RRT estimates to direct questioning. We included RRT design, administration mode, the probability of receiving the sensitive question, whether the RRT and direct question data were collected from the same or different samples, and the type of randomising device used as predictors. All predictors were checked for collinearity prior to modelling. We then assess how well respondents understood the RRT process in each RRT study, and where possible, examine the level of evasive responding.

## 3 Results

### 3.1 Types of study

**Fig. 3:**
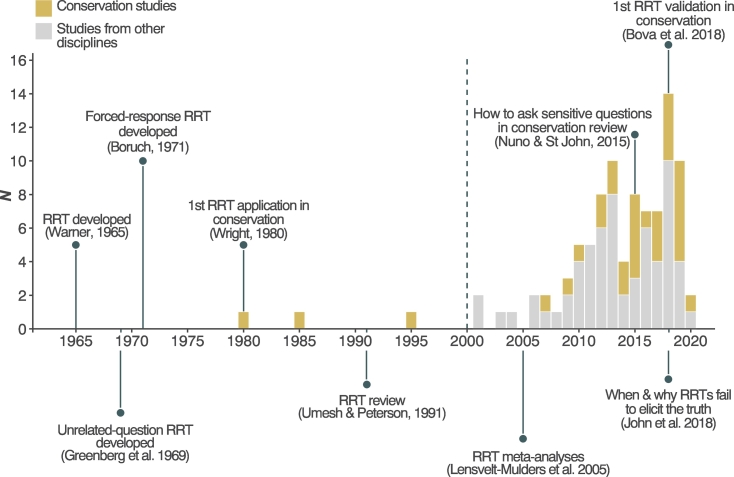
Timeline showing key dates in the development of RRTs, and the number of empirical studies reviewed in this analysis (from conservation and other disciplines) published per year to April 2020. Dashed line indicates the year (2000) after which studies from other disciplines were included (see Methods).

In the 98 studies reviewed, RRT was used to investigate topics including doping in sport (15% of all studies reviewed), sexual behaviour (10%), and drug use (5%) (Fig. B1). We identified 32 studies (33% of all studies reviewed) that used RRT to research conservation topics including illegal hunting of wildlife (44% of conservation studies), breaches of fishing regulations (38%), consumption of wildlife (12%), and illegal extraction of natural resources from protected areas (6%). The first recorded use of RRT in conservation estimated illegal deer hunting in the USA in 1980 (Fig. 3). Across all studies, authors justified the use of an RRT where the topic was illegal or non-compliant (67% of all studies) or involved a socially undesirable behaviour (26%) or view (7%) (Fig. B1). The greatest number of RRT studies were conducted in Germany (24% of all studies), followed by the USA (12%) and UK (8%). Conservation studies were conducted across a wide geographic range; most in the USA (*n* = 4) (Fig. B2).

### 3.2 Data collection approaches

Surveys were predominately administered face-to-face (50% of all studies) or were self-completed (28%) (of which 75% used ballot-boxes to assure additional anonymity), delivered online (21%) or via telephone (3%). Administration mode was not listed in one study, while more than one method was used in four studies. Compared to other disciplines, a greater proportion of conservation surveys were administered face-to-face (87% of conservation studies), with fewer self-completed (15%) or administered online (3%) (Table S2A).

The number of respondents included in each study varied considerably (median = 714, IQR = 298–1862, *n* = 98), with the mean number of respondents significantly higher for studies conducted in other disciplines (median = 1144, IQR = 552–2075, *n* = 66) than conservation (median = 279, IQR = 169–501, *n* = 32) (*t* = −4.628, df = 92.252, *p* = 0.000). Only 28% of studies reported conducting a pilot study or pre-testing the survey instrument prior to data collection.

### 3.3 Variations in RRT design

#### 3.3.1 Design type

**Fig. 4:**
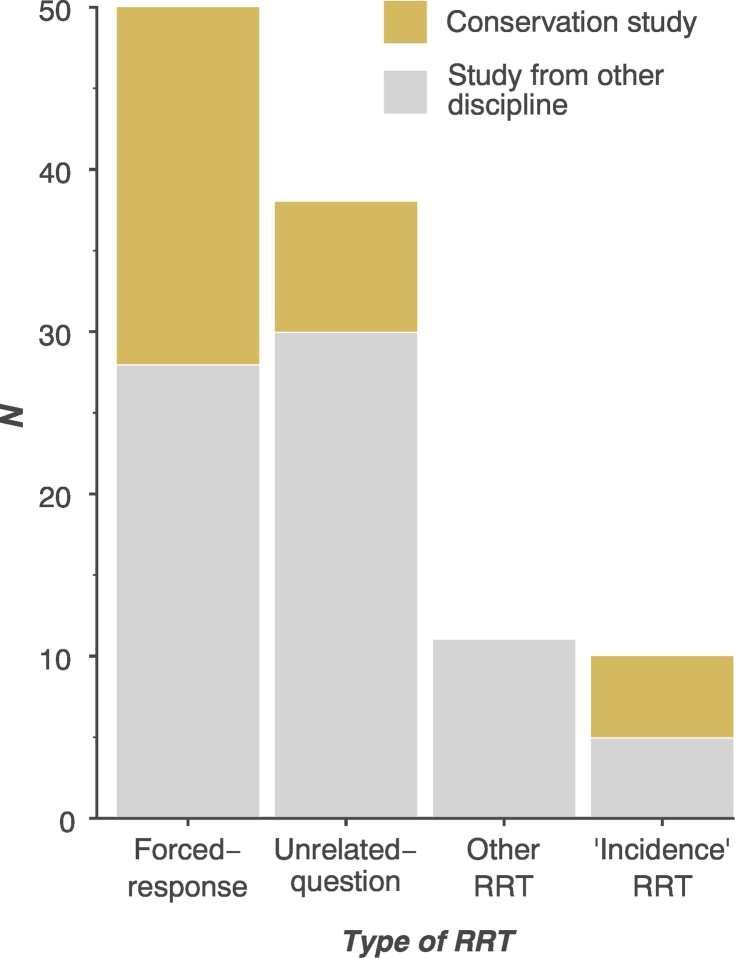
Types of RRT design used. ‘Incidence’ RRT design represents studies which used RRT to estimate frequencies associated with the sensitive characteristic (e.g. additive, or quantative RRT designs).

The most used RRT design was the forced-response (51% of all studies, 69% of conservation studies), followed by the unrelated-question design (including the paired-alternative) (39% of all studies, 25% of conservation studies); ‘incidence’ designs (e.g., an additive, discrete-quantitive or quantitative unrelated-question design) were used in 10% of studies, while 11% adopted other rarely used RRT designs (e.g. multi-group item randomised response (de Jong et al., 2012)) (Fig. 4). Most studies used one RRT design (92%) whilst 5% employed two RRTs, usually to derive different types of estimate (e.g., prevalence and frequency estimates), or to compare different RRT designs. Three studies (3%) used three RRTs. Quantitative or additive RRTs were used in 16% of conservation studies to estimate incidences such as the number of fish caught or number of hunting trips.

#### 3.3.2 Probability of answering the sensitive question

The majority (68%, *n* = 34) of forced-response RRT questions used symmetrical designs, the mean probability of being required to provide a truthful response was 0.72 (min = 0.33, max = 0.9), forced-yes was 0.16 and forced-no was 0.13. In the 32% of studies that used an asymmetric forced-response design, the mean probability of being asked to answer truthfully was lower (0.57, min = 0.5, max = 0.67), and the mean probability of providing a prescribed response, higher (0.44). Within conservation, most studies used a symmetrical forced-response (63% of conservation studies).

For the unrelated-question RRT, the mean probability of receiving the sensitive question was 0.62 (min = 0.5, max = 0.83). Unrelated-question designs used innocuous questions for which the probability was known (74% of unrelated-question studies) and unknown (21%), insufficient detail was provided for two studies. The two most common types of innocuous question with known probabilities asked about a birth date or month, or used a paired-alternative design. This approach was commonly used in (18% of conservation studies).

#### 3.3.3 Randomiser

A variety of randomising devices were used including dice (28% of all studies), coins (16%), birth dates (15%), a ‘lucky dip’ (e.g., counters picked from a container, 13%), tables or lists of numbers which respondents selected from and then matched with electronically generated numbers (11%), deck of cards (9%); 16% used other methods (e.g., Benford's law, free choice, a spinner, numbers listed on bank notes). One study incorrectly conducted randomisation at the group level, rather than individual. No information on the randomising device was provided in one study. Within conservation, the most used devices were dice (47% of conservation studies), ‘lucky dips’ (22%), coins (22%), playing cards (6%) or lists of numbers respondents had to select from (3%).

#### 3.3.4 Number of RRT questions

Respondents were required to answer a mean of five RRT questions per study, 89% of studies asked fewer than 10 RRT questions per respondent, although one study asked 29 RRT questions per respondent. Conservation studies usually asked about multiple forms of rule-breaking within one study, for example, breaches of several different fishing regulations (quotas, fishing gear, fish size), or the killing of several different wildlife species.

### 3.4 How were RRT data analysed?

Most (56%, *n* = 55) studies presented results with confidence intervals (usually at the 95% level), 15% of studies provided standard errors, 4% presented standard deviation, variance was provided but unidentified in 2% of studies, while 27% of studies failed to provide any estimates of variance. To account for the additional uncertainty introduced by the randomising process, 22% of studies reported bootstrapping to derive confidence intervals. Power analyses were conducted prior to data collection in 12% of studies to predict whether the sample would achieve sufficient statistical power. Most studies reported prevalence estimates only (68%), while 31% conducted multivariate analyses, usually using specialised forms of logistic regression or multinomial processing trees to account for noise added by randomisation processes. Prevalence estimates were most often presented in tables (53% of all studies), graphically (32%) or listed in the text (19%). A variety of software was used to analyse data, including R (20% of studies), SPSS (13%), multiTree (4%), or STATA (3%).

### 3.5 Performance of RRTs

RRT estimates were rarely validated using data on known prevalence of sensitive behaviours. Only six studies, published in five articles did so. In these studies, validation data were collected before survey administration (e.g. from government records or covert observation). In one study, RRT overestimated the known prevalence of the sensitive characteristic by 0.2%; but in all other studies RRTs underestimated prevalence (min: 5.9%, max: 55.7%, Table B2). Findings highlight significant variation in RRT performance, and suggest RRTs may be prone to underestimating true prevalence.

**Fig. 5:**
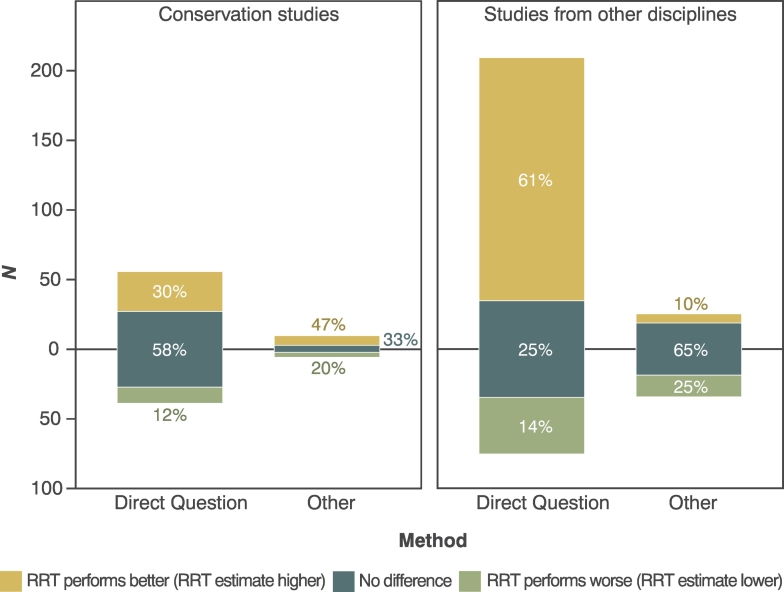
Performance of RRT compared to direct questioning and other specialised questioning techniques (SQT, e.g. the Unmatched Count Technique). Data were available from 43 studies (14 conservation studies, and 29 studies in other disciplines), which asked 319 sensitive questions. Some questions were duplicated (i.e., when more than one method was tested in a study), providing a total of 452 prevalence estimate comparisons.

Nearly half the studies (46% of all studies, *n* = 45) compared RRT estimates to those derived using alternate methods. Most (96%, *n* = 43) compared RRTs to direct questions, while 29% (*n* = 13) compared RRTs against other methods. In conservation, 47% of studies compared RRT estimates against direct questions (93% of conservation studies that compared estimates), or other specialised questioning techniques (16%, e.g. UCT, bean method, false concensus, nominative technique). In other disciplines, RRTs performed better than direct questions across 61% of the questions asked, while in conservation, only 30% of RRT estimates were significantly higher than those of direct questions (Fig. 5). When compared to specialised questioning techniques, a greater proportion of conservation RRT estimates performed better than other disciplines (50% vs. 10%) (Fig. 5). Overall, RRTs provided estimates better than, or equal to (i.e. no significant difference between estimates) those derived using alternate methods the majority of the time (Fig. 5).

Ordered logistic regression suggested RRTs were more likely to secure higher estimates when they allocated a lower probability of answering the sensitive question, used an unrelated-question rather than forced-response design, and responses for each method were collected from separate respondents (rather than respondents answering the same question using two methods). We found no significant effect for randomising device or administration mode (Table 3, Fig. B3).

**Table 3:** *Co-efficient, standard errors, z-values, and p-values from an ordered-logistic regression (with study included as a random-effect), fitted to* assess which factors influence whether RRTs estimates are higher, lower, or indifferent to those derived from direct questions. Comparisons were made between *231 questions* across *32 studies.*

Predictors		Value	SE	z-Value	p-Value
Probability of responding truthfully		**−5.34**	2.30	−2.32	**0.02**⁎
RRT design^a^	Unrelated-question	**1.72**	0.55	3.15	**0.00**⁎⁎
DQ & RRT response from same sample^b^		**−1.27**	0.59	−2.15	**0.03**⁎
Randomising device^c^	Physical (e.g., dice, cards)	1.41	0.89	1.59	0.11
	Virtual (e.g., online spinner)	2.55^·^	1.31	1.95	0.05
Administration mode^d^	Online	−0.19	1.07	−0.18	0.86
	Self-complete & ballot	0.70	1.08	0.65	0.52
	Telephone	−0.34	1.16	−0.30	0.77
RRT performed worse than DQs | No Significant difference ^*e*^		**−4.88**	1.79	−2.74	**0.00**⁎⁎
No significant difference | RRT performed better than DQs		−2.04	1.76	−1.16	0.25
Log Likelihood		−176.46			
AIC		374.92			
BIC		412.64			
Num. obs.		228			
Groups: (Study)		31			
Variance: Study (Intercept)		0.66 (SD: 0.811)			

Notes on reference categories:

^a^Forced-response RRT design.

^b^Direct questions and RRT responses collected from seperate samples.

^c^Personal number randomising device (e.g. birth date or month).

^d^Face-to-face administration mode.

^e^These two rows represent intercepts (cut-points between categories).

⁎⁎ *p* < 0.01.

⁎ *p* < 0.05.

### 3.6 Measuring respondents understanding and adherence to RRT instructions

**Fig. 6:**
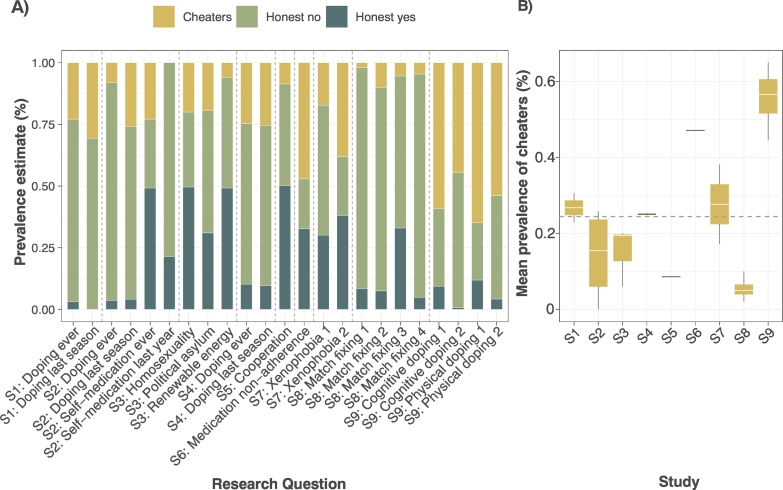
A) Prevalence estimates of the 23 sensitive questions asked in nine studies which used post-hoc analyses (Table S2C) to estimate non-adherence to RRT instructions. Dashed lines indicate questions asked in studies S1-S9. B) Box and whisker plot of cheating prevalence per study; grey dashed line indicates mean estimate of cheating across studies S1-S9 (24.4%). Only one conservation study used post-hoc analyses (Chang et al., 2019), but findings are not included as estimates of cheating were derived across all RRT items, rather than for individual questions.

Overall, respondents' understanding of RRTs was poorly measured and rarely tested. Only 19% of studies (*n* = 19) discussed respondent's understanding of RRTs, of which 58% (*n* = 10) explicitly measured it, usually by asking respondents to identify, on a Likert-type scale, how well they had understood the RRT process. In seven of these studies, high levels of understanding were reported. Numerous studies qualitatively reported that respondents failed to adhere to RRT instructions and instead gave evasive or self-protective responses (e.g. by answering ‘no’ when they were required to provide a forced ‘yes’). Nine studies used post-hoc statistical analyses to detect the proportion of respondents who failed to follow RRT instructions (known as ‘cheating’). Across these studies a mean of 24.4% (min: 0%, max: 64.9%) of responses were thought to be evasive (Fig. 6). In addition, one conservation study (Chang et al., 2019) used item-response theory to estimate cheating in a study of bird hunting. They found 17.5% of all responses did not follow RRT instructions. A further five conservation studies reported that they suspected or knew respondents were failing to adhere to RRT instructions.

Respondents perceptions of the anonymity offered by RRTs was measured in eight studies (8% of all studies), in six of these, most respondents reported they felt RRT increased protection. Only 49% of studies provided respondents assurances of anonymity before starting data collection, while 11% offered confidentiality, although this is likely an underestimate as information on ethical measures was often excluded from manuscripts.

## 4 Discussion

Specialised questioning techniques such as RRTs are increasingly applied in conservation to overcome bias when investigating rule-breaking behaviours such as illegal fishing or hunting. The flexibility of the method, along with positive reviews of their performance suggests RRTs can overcome biases associated with research on sensitive topics. However, our findings, along with reviews by others (Cerri et al., 2021; Lensvelt-Mulders et al., 2005b; Umesh and Peterson, 1991), highlight a need for caution; RRTs do not consistently provide ‘better’ results (Höglinger and Jann, 2018). Validation studies reveal that RRTs typically underestimate true prevalence, and whilst RRTs typically out-perform direct questioning in other fields, our evidence suggests they do not yet do so in conservation. Using information collected throughout our review, we provide advice for conservationists on when RRTs should be used, alongside best practice guidelines when considering RRT design, delivery, and analysis.

### 4.1 When should and shouldn't RRTs be used?

**Fig. 7:**
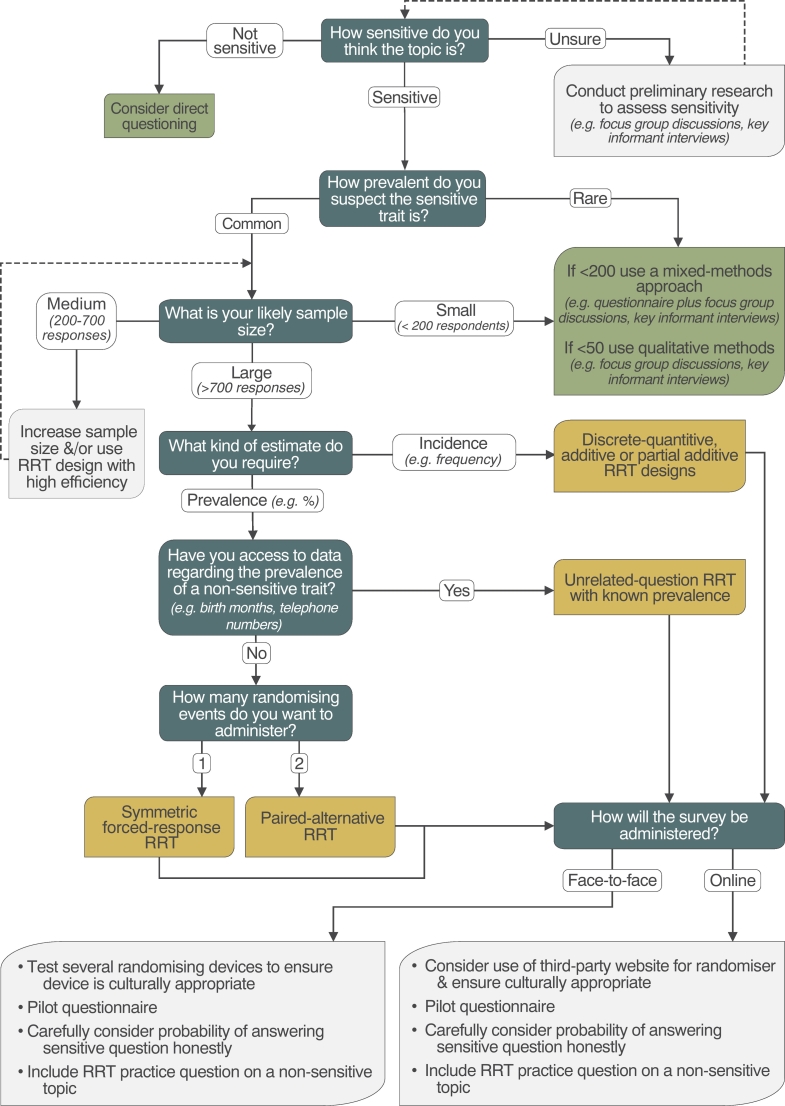
Decision tree to identify a) whether an RRT is appropriate (blue boxes), b) the most suitable RRT design (green boxes), and c) considerations to improve robustness (light grey boxes). (For interpretation of the references to colour in this figure legend, the reader is referred to the web version of this article.)

Conservationists often investigate behaviours that involve endangered species or rare resources. An inherent reason why these are of conservation interest is due to their declining abundance, thus the prevalence of these behaviours is also likely to be scarce. Randomised response procedures add noise to data, meaning estimates suffer large standard errors, and reduced power (Lensvelt-Mulders et al., 2005b), as a result behaviours which are exceptionally rare can yield inconclusive results (for example, see St John et al., 2018). While increasing sample sizes can overcome this, often this comes at additional cost (e.g., time, money), or may be impossible if the target population is small. Thus, if researching behaviours that are predicted to be rare, and/or it is only possible to achieve a small sample size, qualitative methods, such as key informant interviews, may be more suitable (Davis et al., 2020). Before deciding whether to use RRT, or indeed any specialised questioning technique, we recommend consideration of a range of factors, including how sensitive the topic is, the likely sample size and the type of estimate required (e.g., prevalence in the population, or an estimate of incidence) (Fig. 7).

Having committed to incorporating RRT into a study, researchers must make decisions about RRT design and administration. The forced-response RRT and unrelated-question RRT have been identified as the most efficient designs (Lensvelt-Mulders et al., 2005a), while our model suggested the unrelated-question (including the paired-alternative design) was better at reducing bias. However, there are elements of both designs that can be adjusted on a case-by-case basis to improve performance. These include the probability of respondents answering truthfully (*p*), and the type of randomising device used. The closer *p* is to 1, the more efficient the design, and the smaller the sample size required (Fox, 2016). However, as demonstrated in our model, allocating a *p* value too high undermines the protection offered by the method, and can discourage truthful responding; set too low, and the number of affirmative responses may be insufficient to produce robust estimates. Research suggests the optimal value for *p* lies between 0.75 and 0.8 (Soeken and Macready, 1982). Identifying a suitable randomising device is key. Ideally, randomisers should be simple, familiar, easy to use and importantly, trusted by respondents. Be aware, in some contexts, devices may have undesirable conations, for example, when investigating bushmeat consumption in Madagascar Razafimanahaka et al. (2012) reported dice were associated with gambling. Consequently, they utilised a ‘lucky dip’ format and asked respondents to select different coloured balls from a bag. Moreover, when conducting experimental research to assess virtual/online randomisers, Coutts and Jann (2011) found automated randomisers were trusted less due to concerns of anonymity and randomiser manipulation. Directing participants to third-party websites can overcome this. For example, in their study of marijuana use, Cobo et al. (2017) encouraged respondents to download an independent card app which respondents used to randomly select a card from a deck and determine the answer they should give. This approach requires care to ensure randomising outcomes are not suruptiously recorded by the website, as this would count as deceptive research with ethical implications. Testing several randomisers before data collection, paying close attention to how each device is received and asking respondents for feedback will ensure an appropriate device is chosen.

The type of randomiser used is also influenced by how surveys are delivered. Research has shown online response times can be quicker when using automated devices (e.g., electronic coin toss), and that devices that require shifts away from the survey mode (i.e., locating and manually tossing a coin) can induce higher levels of non-response (Coutts and Jann, 2011). Making small tweaks to how randomisers are used can improve design efficiency. For example, using two dice (instead of one) and asking respondents to sum scores together, enables researchers to capitalise on people's poor calculations of probability, and also provides respondents with an augmented sense of protection (Cross et al., 2013). If asked to provide a truthful response when 5–10 is scored, a respondent may believe they have a 0.5 chance of providing an honest response, yet in reality they will roll a truthful score 75% of the time (Cross et al., 2013; Lensvelt-Mulders et al., 2005b). However, summing the scores of two dice together adds another step to the response process and may increase cognitive load. For devices other than dice, efficiency is improved more easily, for example, adding extra cards to a deck, or counters to a bag, increases the *p* but without increasing cognitive burden.

As with all methods, successful implementation depends upon rigorous piloting; for RRT, this includes trialling the script introducing RRT and the equipment. Multiple rounds of piloting may be required if issues are detected (Newing, 2011). Presenting the method as ‘being like a game’ with ‘rules to follow’, can help (St John et al., 2012; Razafimanahaka et al., 2012)**,** as can practice questions about non-sensitive topics. These help familiarise respondents with RRT processes and could involve role reversal, enabling participants to experience the process from enumerators' perspectives (St John et al., 2015). To study bird hunting in China, Chang et al. (2019) asked two training questions about common behaviours (“Do you play cards?”, “Do you drink [alcohol]?”) before sensitive questions to ensure respondents understood. Repeating this process until the enumerator is confident the respondent understands the process is important. If pre-tests indicate respondent concerns regarding privacy, consider mitigating these using additional measures (e.g. using a ballot-box if surveys are self-administered, revising the randomising device, reducing *p*) (Arias et al., 2020; Krumpal and Voss, 2020). If understanding is not reached, it is useful to provide enumerators with a mechanism to record this, so that potentially confused responses can be excluded from analysis. As with all research, who the enumerator is, is important. In Madagascar, Razafimanahaka et al. (2012) found recruiting someone from the same community to help explain RRTs to participants invaluable. They found that even though trained enumerators spoke local dialects, seeing a familiar person who was clearly comfortable with the method gave respondents the confidence to engage with it.

Small changes in how responses options are phrased can also impact results. During a series of online experiments, John et al. (2018) found that adapting the forced-response answer respondents were required to give, resulted in more accurate prevalence estimates compared to standard forced-responses. For example, changing binary “yes” or “no” responses to “yes, or flipped heads” or “no” increased the ambiguity of the response, and emphasized to respondents that "yes" meant “yes, I do the sensitive behaviour” and “yes, I flipped a head”. The effect was strongest amongst respondents who did not possess the sensitive characteristic but were forced to respond affirmatively, this group were more likely to follow instructions when using the revised forced-response RRT. Interestingly, the effect became more pronounced when anonymity was assured, with the revised-RRT providing higher estimates than a normal forced-response RRT (John et al., 2018). Considering how instructions are delivered can also be effective. Instead of stating *“you must say yes”,* greater responding may be encouraged by acknowledging that answers may be contrary to the truth, for example by saying, *“if your dice lands on 6, you simply have to answer yes, even if this is not your true answer”.*

Our findings highlight that unlike other disciplines, most conservation RRTs are delivered face-to-face. Often this is because research is conducted in contexts where illiteracy is high and access to technology low. However, the uptake of non-face-to-face enumeration modes (i.e. online) will likely increase in conservation, particularly during the Covid-19 pandemic, and as technological access improves and the need to better understand behaviours and attitudes of those engaged in controversial topics (e.g. consumption of illegal wildlife products, trophy hunting) increases. Unlike face-to-face administration, it is more challenging to provide respondents with tailored assistance when delivering surveys online. If respondents do not comprehend how RRT protects them, levels of self-protective answering may rise, especially if a forced-response RRT design is used (Höglinger et al., 2016). Careful thought and extensive pre-testing will help detect this. Providing respondents with clear, and culturally appropriate information about the research and how the data will be used is essential, and should reassure concerned participants (Ibbett and Brittain, 2020). Consent to participate should be given freely, and in return respondents should be provided with assurances of anonymity and confidentiality. Not only does this ensure ethical integrity (Brittain et al., 2020), but research suggests it can reduce bias (Ong and Weiss, 2000).

Researchers often wish to understand which variables best characterise those who possess sensitive traits by conducting multi-variate analyses. However, due to the random noise added to RRT, specialised forms of analysis are required (Keane et al., 2015). Several software packages have been developed for this purpose. The R package ‘rr’ (Blair et al., 2015) enables logistic regression for four RRT designs as well as univariate power analyses, while the package ‘RRreg’ goes further and provides logistic and linear regression models for a large class of randomised response designs (Heck and Moshagen, 2018). Analysis at the individual level can also be conducted by combining randomised-response approaches with item-response theory (Fox and Meijer, 2008). Chang et al. (2018) developed an R package specifically for conservationists adopting this approach. ‘zapstRR’ includes code for univariate analysis of multiple behaviours (e.g. hunting more than one species), methods for estimating the total prevalence of the sensitive behaviour across all RRT questions (known as Sum Scores), plus code to estimate evasive-response bias (Chang et al., 2018). In addition, Cerri et al. (2018) provide R code for multi-variate analyses of RRTs with polychotomous response options. Multinomial processing-tree models, which involve approaches applied in psychology to model observed categorical frequencies as a function of a sequence of latent states can also be employed using ‘multiTree’ software (Moshagen, 2010).

Overall, our understanding of the ability of RRTs to reduce biases is hampered by too few validation studies. The only conservation study to validate findings was Bova et al. (2018), who covertly observed fishers and later questioned those who breached regulations about their behaviour using RRT and direct questioning with ballot-box. Replicating this approach is challenging; behaviours often occur in secret (e.g. illegal hunting), in places difficult to observe (e.g. in dense forest), may place researchers and respondents at risk, and can raise ethical questions about the role of research. Wherever possible, multiple sources of data (e.g., key informant interviews, arrest records, previous studies) should be used to triangulate and corroborate findings from RRT studies. In conservation, there is a tendency to compare RRT data by asking respondents the same questions using different methods, however, this undermines the protection provided by RRTs (particularly if direct questions are used), can erode trust, and contribute to survey fatigue (Ibbett and Brittain, 2020). In other disciplines, best practice is to collect data from separate samples using different methods, ideally adjusted at a ratio of 2:1, where two RRT responses are collected for every one direct question response (Razafimanahaka et al., 2012), our model also suggests this approach provides higher RRT estimates.

In other disciplines, experiments are increasingly applied to assess respondents comprehension and willingness to follow RRT instructions (Hoffmann et al., 2017; John et al., 2018)***,*** such approaches have not yet been applied in conservation but would be informative. Amendments to RRT design and post-hoc analyses can also help to determine the proportion of respondents following RRT instructions. For example, the Cheating Detection Model developed by Clark and Desharnais (1998) is designed to quantify the extent of non-adherence to RRT instructions (Fig. S2D). Ostapczuk et al. (2011) used this approach to estimate the proportion of patients failing to take medication prescribed by their physician. Recently, the model has been extended to incorporate multiple RRT questions (Multiple issues cheating model, Moshagen and Musch, 2012) and for use with unrelated-question RRT designs (Reiber et al., 2020). Advances also aim to account for situations where social desirability does not occur in the assumed direction. The no-cheater detection and total-cheater detection models aim to improve estimates of evasive responding under these scenarios (Feth et al., 2017). Applications of these variations remain rare in conservation (but see Chang et al., 2019), yet use would enhance researcher's ability to assess the reliability of RRT data.

## 5 Conclusion

Our review demonstrates that RRTs have become an important tool for conservation researchers investigating sensitive topics. To date, they have been predominately applied in face-to-face research to quantify the incidence or prevalence of non-compliant behaviour, such as illegal consumption of wildlife, or breaching of fishing regulations. Within conservation, there is increasing recognition of the need to understand human behaviour (Cinner, 2018) and in light of Covid-19, there is likely to be a shift towards more online data collection (Wardropper et al., 2021). Methods that can reduce bias when asking sensitive questions, which can be administered in multiple ways, are an valuable addition to the research toolbox. With more accurate data, conservationists can better target, and better evaluate the impact of interventions aimed at reducing rule-breaking (St John et al., 2013). By following our detailed guidance, conservation researchers can firstly assess whether an RRT is appropriate, and secondly, develop more robust research designs. We strongly emphasize that to be successful, RRT studies require careful piloting and a strong understanding of their strengths and limitations, as well as the context in which the study will occur.

## Declaration of competing interest

The authors declare that they have no known competing financial interests or personal relationships that could have appeared to influence the work reported in this paper.

## Appendix A Supplementary data

The search protocol, a list of all studies reviewed and some additional results can be found in the supplmentary materials. Image 1
