# Supplementary material for: Asking sensitive questions in conservation using Randomised Response Techniques
Source: Biol Conserv. 2021 Aug;260:109191. doi: 10.1016/j.biocon.2021.109191 (PMC8346952; doi:10.1016/j.biocon.2021.109191)
Supplement: Supplementary file 1 — The search protocol, a list of all studies reviewed and some additional results can be found in the supplmentary materials. [file mmc1.docx]

**Appendices**

**Appendix A – Review Structure & data extraction protocol**

**Stage 1 – Systematically search databases**

**Databases:** Web of Science, Scopus

**Search term:** "Randomised Response Technique" OR "Randomized Response Technique”

*Note // The acronym RRT was removed from the search term on the assumption that any article that used an acronym would also use the full term. In addition, RRT has alternate definitions in other disciplines (e.g. Rapid Response Team in medicine), thus removing the acronym avoided a proliferation of irrelevant search results.*

**Document type**: Peer-reviewed Articles only

**Language:** English only

**Research areas/categories:** All

**Dates:** 1970 to 2020

**Recorded:** date of search, database, search terms & selected criteria, number of results, researcher conducting the search.

Extract .csv of all search results and import into excel. For each .csv of search results:

1. Add column and allocate each paper an ID code, identifying which database the article was retrieved from, and the number in the list (e.g. the first record from Scopus coded as = Sco_01);
2. Combine all the search results into one spreadsheet;
3. Re-sort the rows alphabetically by author, then article title; and
4. Use the conditional formatting function to identify duplicates, mark duplicates, ensure only one version of article left in list.

**Stage 2 – Title Scan**

Does the does title mention or suggest use of RRT?

- If Yes, forward for abstract review.
- If unsure, forward for abstract review.
- If No, exclude.

**Stage 3 – Abstract Scan**

Does the abstract mention use of RRT to collect empirical data on a sensitive topic?

- If Yes, forward for full review.
- If unsure, forward for full review.
- If No, exclude.

**Stages 4 & 5 – Data Extraction**

Read the full article, and extract the following information:

- Article details (Authors, Title, Journal, DOI, Year published, Academic discipline);
- Study details (Behaviour(s) studied, sensitivity of behaviour ((a) non-compliant or illegal behaviour (e.g. smuggling); b) socially undesirable behaviour (e.g. promiscuity); c) socially undesirable views (e.g. racism); d) socially desirable behaviour (e.g. recycling); e) personal or health topic (e.g. being HIV positive)), study country, year data collected, sample size, number of RRT questions);
- Methods details (RRT design, description of RRT method, administration mode, probability of truthful, forced-yes (or unrelated question) and forced-no responses, randomising device used, prompts used, whether a practice question and/or control question asked, whether a pilot was conducted);
- Validation details (Were RRT estimates compared against data on known prevalence, or estimates derived using other questioning methods (e.g. direct questions, other SQTs). Record details;
- Analysis details (Were power analyses conducted? What type of analyses were conducted (unknown, univariate, multivariate), software and packages used);
- Results details (prevalence estimate, type of error presented (i.e. SE, 95% CI, none), were RRT estimates higher, lower or non-statistically different than those collected using other methods, Was understanding measured and findings, was whether respondents followed instructions assessed? Findings. Was privacy measured and findings. Was RRT reported as successful. How were results presented (e.g. graph, table));
- Ethics details (Did the article state whether**:** research was approved by an ethics board; free, prior and informed consent was sought from participants; participants were provided assurances of anonymity and/or confidentiality?).

Figure A1. Flow diagram showing the inclusion criteria and structure of the systematic review according to the Reporting standards for Systematic Evidence Synthesis (ROSES) from Haddaway NR, Macura B, Whaley P, and Pullin AS. 2017. ROSES flow diagram for systematic reviews. Version 1.0. DOI: 10.6084/m9.figshare.5897389

Table A1. List of 95 articles included in review (n=98 studies)

| **#** | **Authors** | **Title & DOI** | **Journal** |
| --- | --- | --- | --- |
| *Conservation Studies* | | | |
| 1 | Akinsorotan et al. 2019 | Evaluating Rule Breaking Behavior in a Nigerian Protected Forest Reserve Area | Journal of Applied Science and Environmental Management |
| 2 | Arias & Sutton, 2013 | Understanding recreational fishers' compliance with no-take zones in the Great Barrier Reef Marine Park | Ecology and Society |
| 3 | Blank & Gavin 2009 | The randomized response technique as a tool for estimating non-compliance rates in fisheries: A case study of illegal red abalone *(Haliotis rufescens)* fishing in Northern California | Environmental Conservation |
| 4 | Bova et al. 2018. | Limitations of the random response technique and a call to implement the ballot box method for estimating recreational angler compliance using surveys | Fisheries Research |
| 5 | Carvalho et al. 2019 | Jaguar hunting in Amazonian extractive reserves: acceptance and prevalence | Environmental Conservation |
| 6 | Castilho et al. 2019 | Hunting of mammal species in protected areas of the southern Bahian Atlantic Forest, Brazil | Oryx |
| 7 | Cerri et al 2017 | The randomised response technique: A valuable approach to monitor pathways of aquatic biological invasions | Fisheries Management and Ecology |
| 8 | Cerri et al. 2017 | Are wildlife value orientations useful tools to explain tolerance and illegal killing of wildlife by farmers in response to crop damage? | European Journal of Wildlife Research |
| 9 | Chaloupka, 1985 | Application of the randomized response technique to marine park management: an assessment of permit compliance | Environmental Management |
| 10 | Chang et al. 2019 | Perceived entertainment and recreational value motivate illegal hunting in Southwest China | Biological Conservation |
| 11 | Conteh & Gavin, 2017 | Influence of war on hunting patterns and pressure in Sierra Leone | Environmental Conservation |
| 12 | Conteh et al. 2015 | Quantifying illegal hunting: A novel application of the quantitative randomised response technique | Biological Conservation |
| 13 | Craig et al. 2019 | The drivers and extent of poison use by Namibia's communal farmers: Implications for averting the African vulture crisis | Ambio |
| 14 | Cross et al. 2013 | Innovative Techniques for Estimating Illegal Activities in a Human-Wildlife-Management Conflict | PLoS ONE |
| 15 | Davis et al. 2019 | Understanding the prevalence of bear part consumption in Cambodia: A comparison of specialised questioning techniques | PLoS ONE |
| 16 | Gálvez et al. 2018 | A spatially integrated framework for assessing socioecological drivers of carnivore decline | Journal of Applied Ecology |
| 17 | Lancaster et al. 2015 | Drivers of recreational fisher compliance in temperate MCAs | Global Ecology and Conservation |
| 18 | Lewis, 2015 | Bags and tags: Randomized response technique indicates reductions in illegal recreational fishing of red abalone *(Haliotis rufescens)* in Northern California | Biological Conservation |
| 19 | Oyanedel et al. 2018 | Illegal fishing and territorial user rights in Chile | Conservation Biology |
| 20 | Oyanedel et al. 2020 | Motivations for (non‐)compliance with conservation rules by small‐scale resource users | Conservation Letters |
| 21 | Randriamamonjy et al. 2015 | Consumption of bushmeat around a major mine, and matched communities, in Madagascar | Biological Conservation |
| 22 | Razafimanahaka et al. 2012 | Novel approach for quantifying illegal bushmeat consumption reveals high consumption of protected species in Madagascar | Oryx |
| 23 | Robinson et al. 2015 | Captive reptile mortality rates in the home and implications for the wildlife trade | PLoS ONE |
| 24 | Santangeli et al. 2016 | Understanding, quantifying and mapping the use of poison by commercial farmers in Namibia – Implications for scavengers' conservation and ecosystem health | Biological Conservation |
| 25 | Schill & Kline, 1995 | Use of Randomised Response to Estimate Angler Noncompliance with fishing regulations | North American Journal of Fisheries Management |
| 26 | Solomon et al. 2007 | Estimating illegal resource use at a Ugandan park with the randomized response technique | Human Dimensions of Wildlife |
| 27 | St John et al. 2012 | Identifying indicators of illegal behaviour: Carnivore killing in human-managed landscapes | Proceedings of the Royal Society B: Biological Sciences |
| 28 | St John et al. 2018 | Intention to kill: Tolerance and illegal persecution of Sumatran tigers and sympatric species | Conservation Letters |
| 29 | St. John et al. 2010 | Testing novel methods for assessing rule breaking in conservation | Biological Conservation |
| 30 | St. John et al. 2014 | Evaluating deterrents of illegal behaviour in conservation: Carnivore killing in rural Taiwan | Biological Conservation |
| 31 | Thomas et al. 2014 | Estimating non-compliance among recreational fishers: Insights into factors affecting the usefulness of the randomized response and item count techniques | Biological Conservation |
| 32 | Wright, 1980 | Use of Randomized Response Technique to Estimate Deer Poaching | Wildlife Society Bulletin |
|  | *Studies from other disciplines* | | |
| 33 | Akwataghibe et al. 2013 | Assessing health workers' revenues and coping strategies in Nigeria - A mixed-methods study | BMC Health Services Research |
| 34 | Anglewicz et al. 2013 | The effect of interview method on self-reported sexual behavior and perceptions of community norms in Botswana | AIDS and Behavior |
| 35 | Bailey et al. 2001 | Research misconduct in accounting literature: A survey of the most prolific researchers’ actions and beliefs | Abacus |
| 36 | Boardley et al. 2019 | Perceptions of coach doping confrontation efficacy and athlete susceptibility to intentional and inadvertent doping | Scandinavian Journal of Science and Medicine in Sports |
| 37 | Chen et al. 2014 | The randomized response technique application in the survey of homosexual commercial sex among men in Beijing | Iranian Journal of Public Health |
| 38 | Chong et al. 2019 | Asking Sensitive Questions Using the Randomized Response Approach in Public Health Research: An Empirical Study on the Factors of Illegal Waste Disposal | International Journal of Environmental Research and Public Health |
| 39 | Chu et al. 2018 | Applying the Randomized Response Technique in Business Ethics Research: The Misuse of Information Systems Resources in the Workplace | Journal of Business Ethics |
| 40 | Cobo et al. 2017 | Application of randomized response techniques for investigating cannabis use by Spanish university students | International Journal of Methods in Psychiatric Research |
| 41 | Coutts & Jann, 2011 | Sensitive questions in online surveys: Experimental results for the randomized response technique (RRT) and the unmatched count technique (UCT) | Sociological Methods and Research |
| 42 | Cross et al. 2010 | Use of a Randomized Response Technique to obtain sensitive information on animal disease prevalence | Preventive Veterinary Medicine |
| 43 | De Jong et al. 2010 | Reducing social desirability bias through item randomized response: An application to measure underreported desires | Journal of Marketing Research |
| 44 | De Jong et al. 2012 | Analysis of sensitive questions across cultures: An application of multigroup item randomized response theory to sexual attitudes and behavior | Journal of Personality and Social Psychology |
| 45 | Dietz et al. 2013 | Randomized response estimates for the 12-month prevalence of cognitive-enhancing drug use in university students | Pharmacotherapy |
| 46 | Dietz et al. 2013 | Associations between physical and cognitive doping - A cross-sectional study in 2.997 triathletes | PLoS ONE |
| 47 | Dietz et al. 2016 | Analgesics use in competitive triathletes: its relationship to doping and on predicting its usage | Journal of Sports Sciences |
| 48 | Dietz et al. 2018 | Physical and cognitive doping in university students using the unrelated question model (UQM): Assessing the influence of the probability of receiving the sensitive question on prevalence estimation | PLoS ONE |
| 49 | Donovan et al. 2003 | An assessment of the prevalence, severity, and verifiability of entry-level applicant faking using the randomized response technique | Human Performance |
| 50 | Elbe & Pitsch, 2018 | Doping prevalence among Danish elite athletes | Performance Enhancement and Health |
| 51 | Fox et al. 2013 | Mixture randomized item-response modeling: A smoking behavior validation study | Statistics in Medicine |
| 52 | Franke et al. 2013 | Use of illicit and prescription drugs for cognitive or mood enhancement among surgeons | BMC Medicine |
| 53 | Franzen & Pointner, 2012 | Anonymity in the dictator game revisited | Journal of Economic Behavior and Organization |
| 54 | Frenger et al. 2016 E. | Sport-induced substance use-an empirical study to the extent within a German Sports Association | PLoS ONE |
| 55 | Frenger et al. 2019 | Corruption in Olympic Sports: Prevalence Estimations of Match Fixing Among German Squad Athletes | Sage Open |
| 56 | Geng et al. 2016. | Behavioral risk profile of men who have sex with men in beijing, China: Results from a cross‑sectional survey with randomized response techniques | Chinese Medical Journal |
| 57 | Ghofrani et al. 2018 | Prevalence of Induced Abortion in Iran: A Comparison of Two Indirect Estimation Techniques | International perspectives on sexual and reproductive health |
| 58 | Hejri et al. 2013 | Academic disintegrity among medical students: A randomised response technique study | Medical Education |
| 59 | Hilbig et al. 2015 | Truth Will Out: Linking Personality, Morality, and Honesty Through Indirect Questioning | Social Psychological and Personality Science |
| 60 | Hoglinger & Jann, 2018 | More is not always better: An experimental individual-level validation of the randomized response technique and the crosswise model | PLoS ONE |
| 61 | Höglinger et al. 2016 | Sensitive questions in online surveys: An experimental evaluation of different implementations of the randomized response technique and the crosswise model | Survey Research Methods |
| 62 | Husain et al. 2018 | How much self-presentation behavior do applicants from the United Arab Emirates exhibit? | International Journal of Selection and Assessment |
| 63 | Jing et al. 2018 | Combining the randomized response technique and the network scale-up method to estimate the female sex worker population size: an exploratory study | Public Health |
| 64 | Jones et al. 2017 D. | Estimating the prevalence of food risk increasing behaviours in UK kitchens | PLoS ONE |
| 65 | Khadem-Rezaiyan & Dadgarmoghaddam, 2017 | Research misconduct: A report from a developing country | Iranian Journal of Public Health |
| 66 | Kirchner, 2015 | Validating sensitive questions: A comparison of survey and register data | Journal of Official Statistics |
| 67 | Kirtadze et al. 2018 | Republic of Georgia estimates for prevalence of drug use: Randomized response techniques suggest under-estimation | Drug and Alcohol Dependence |
| 68 | Koenig et al. 2012 | How Much do Chinese Applicants Fake? | International Journal of Selection and Assessment |
| 69 | König et al. 2011 | Applicants' Self-presentational Behavior across Cultures: Less self-presentation in Switzerland and Iceland than in the United States | International Journal of Selection and Assessment |
| 70 | Krumpal, 2012 | Estimating the prevalence of xenophobia and anti-Semitism in Germany: A comparison of randomized response and direct questioning | Social Science Research |
| 71 | Kwan et al. 2010 | Applying the randomized response technique to elicit truthful responses to sensitive questions in is research: The case of software piracy behavior | Information Systems Research |
| 72 | Lara et al. 2004 | Measuring Induced Abortion in Mexico: A Comparison of Four Methodologies | Sociological Methods and Research |
| 73 | Lara et al. 2006. | The measure of induced abortion levels in Mexico using random response technique | Sociological Methods and Research |
| 74 | Lavender & Anderson, 2009 | Effect of perceived anonymity in assessments of eating disordered behaviors and attitudes | International Journal of Eating Disorders |
| 75 | Miner & Center, 2008 | Improving the measurement of criminal sexual behavior: The application of randomized responding technique | Sexual Abuse: Journal of Research and Treatment |
| 76 | Moshagen & Musch, 2012 | Surveying multiple sensitive attributes using an extension of the randomized-response technique | International Journal of Public Opinion Research |
| 77 | Moshagen et al. 2011 | Defection in the dark? A randomized-response investigation of cooperativeness in social dilemma games | European Journal of Social Psychology |
| 78 | Ostapczuk et al. 2009. | A randomized-response investigation of the education effect in attitudes towards foreigners | European Journal of Social Psychology |
| 79 | Ostapczuk et al. 2011 | Improving self-report measures of medication non-adherence using a cheating detection extension of the randomised-response-technique | Statistical Methods in Medical Research |
| 80 | Perri et al. 2018 | A mixed-mode sensitive research on cannabis use and sexual addiction: improving self-reporting by means of indirect questioning techniques | Quality and Quantity |
| 81 | Petróczi et al. 2011 | New non-randomised model to assess the prevalence of discriminating behaviour: A pilot study on mephedrone | Substance Abuse: Treatment, Prevention, and Policy |
| 82 | Pitsch & Emrich, 2012 | The frequency of doping in elite sport: Results of a replication study | International Review for the Sociology of Sport |
| 83 | Pitsch et al. 2007 | Doping in elite sports in Germany: results of a www survey | European Journal for Sport and Society |
| 84 | Robertson & Rymon, 2001 | Purchasing agents' deceptive behavior: A randomized response technique study | Business Ethics Quarterly |
| 85 | Rosenfeld et al. 2016 | An Empirical Validation Study of Popular Survey Methodologies for Sensitive Questions | American Journal of Political Science |
| 86 | Rueda et al. 2020 | Measuring Inappropriate Sexual Behavior Among University Students: Using the Randomized Response Technique to Enhance Self-Reporting | Sexual Abuse: Journal of Research and Treatment |
| 87 | Schröter et al. 2016 | A comparison of the cheater detection and the unrelated question models: A randomized response survey on physical and cognitive doping in recreational triathletes | PLoS ONE |
| 88 | Seifarth et al. 2019 | The Prevalence of Legal Performance-Enhancing Substance Use and Potential Cognitive and or Physical Doping in German Recreational Triathletes, Assessed via the Randomised Response Technique | Sports |
| 89 | Simon et al. 2006 | Doping in fitness sports: Estimated number of unreported cases and individual probability of doping | Addiction |
| 90 | Srivastava et al. 2015 | Application of Randomized Response Techniques in Estimation of prevalence of Child Sexual Abuse | Statistics and Applications |
| 91 | Striegel et al. 2010 | Randomized response estimates for doping and illicit drug use in elite athletes | Drug and Alcohol Dependence |
| 92 | Stubbe et al. 2014. | Prevalence of use of performance enhancing drugs by fitness centre members | Drug Testing and Analysis |
| 93 | Tu & Hsieh, 2017 | Estimates of Lifetime Extradyadic Sex Using a Hybrid of Randomized Response Technique and Crosswise Design | Archives of Sexual Behavior |
| 94 | Ulrich et al. 2018 | Doping in Two Elite Athletics Competitions Assessed by Randomized-Response Surveys | Sports Medicine |
| 95 | Wolter & Preisendörfer, 2013 | Asking Sensitive Questions: An Evaluation of the Randomized Response Technique Versus Direct Questioning Using Individual Validation Data | Sociological Methods and Research |

**Appendix B – Additional Figures & Tables**

Additional figures and tables complimentary to those in the main manuscript.


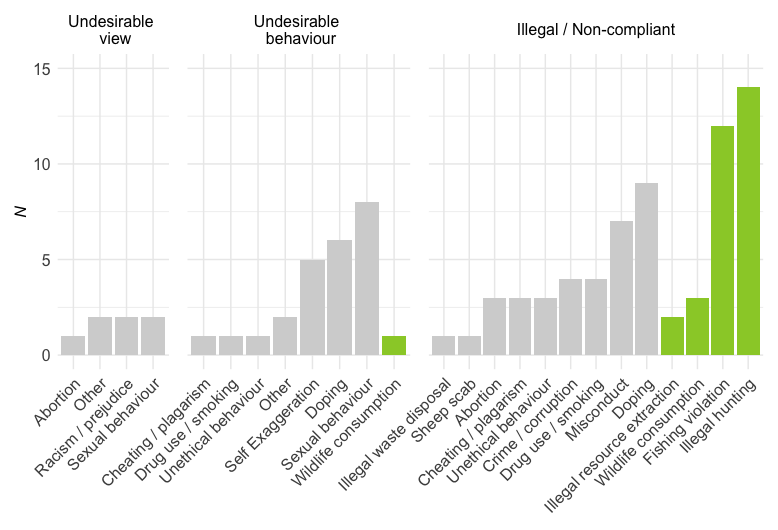


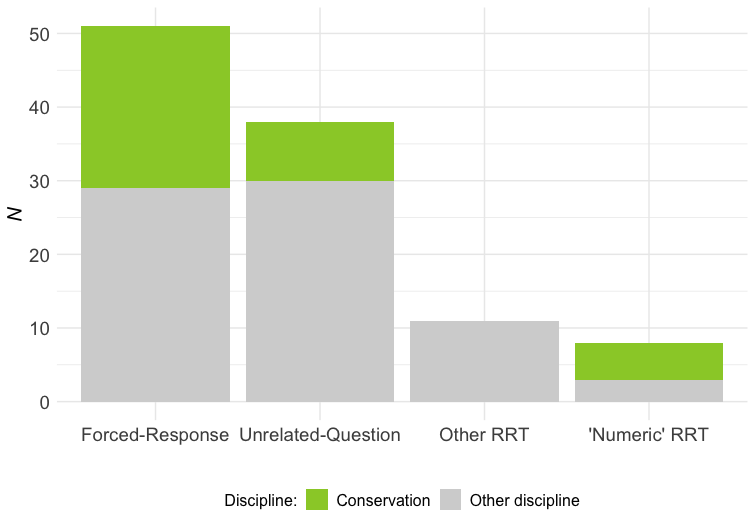


Figure B1. Behaviours RRT was used to investigate, categorised in ascending order by the sensitivity of the research topic


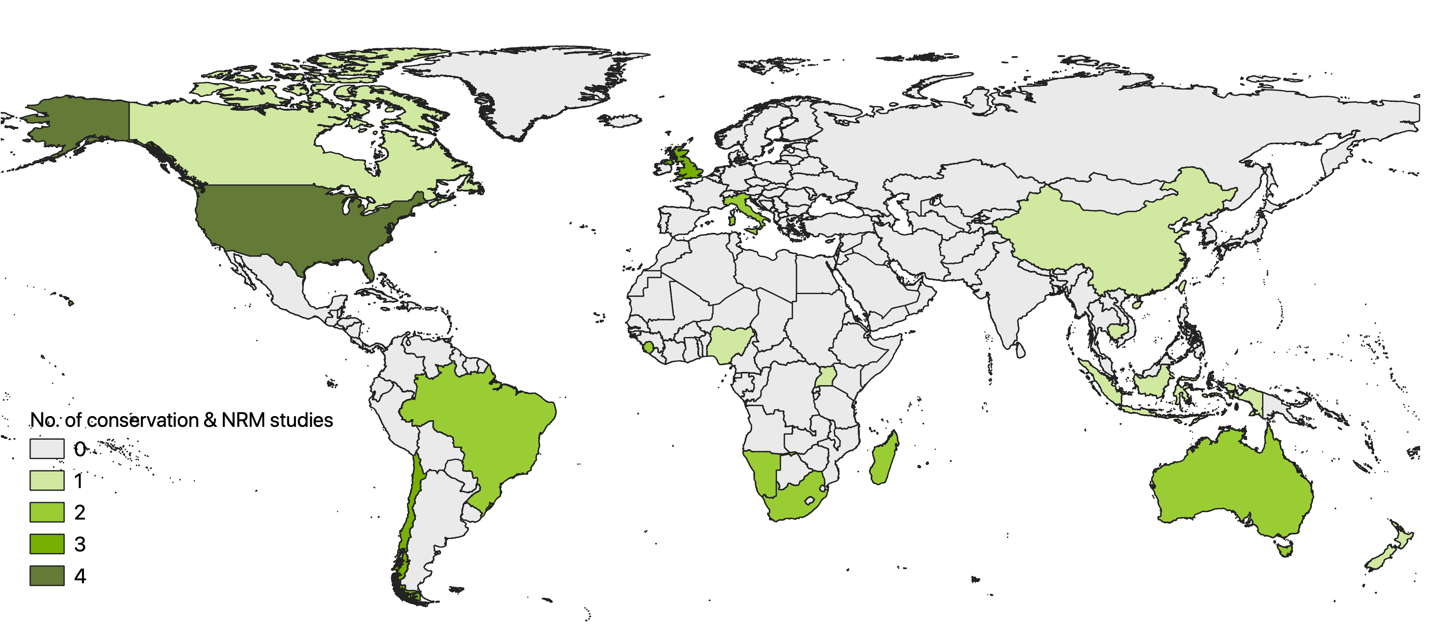


Figure B2. Geographical distribution of all known peer-reviewed studies conducted in conservation that used an RRT (n=32).

Table B1. Modes of administering RRT surveys, displayed by discipline. Four studies used >1 administration mode.

| Administration Method | Conservation | | Other disciplines | | All studies | |
| --- | --- | --- | --- | --- | --- | --- |
|  | (n=32) | % | (n=66) | % | (n=98) | % |
| Face-to-face | 28 | 87% | 22 | 33% | 50 | 50% |
| Self-complete | 5 | 15% | 22 | 33% | 27 | 28% |
| …. with ballot box | 3 | 9% | 17 | 26% | 20 | 21% |
| Online | 1 | 3% | 20 | 30% | 21 | 21% |
| Telephone | 0 | 0% | 3 | 5% | 3 | 3% |
| Unknown | 0 | 0% | 1 | 1% | 1 | 1% |


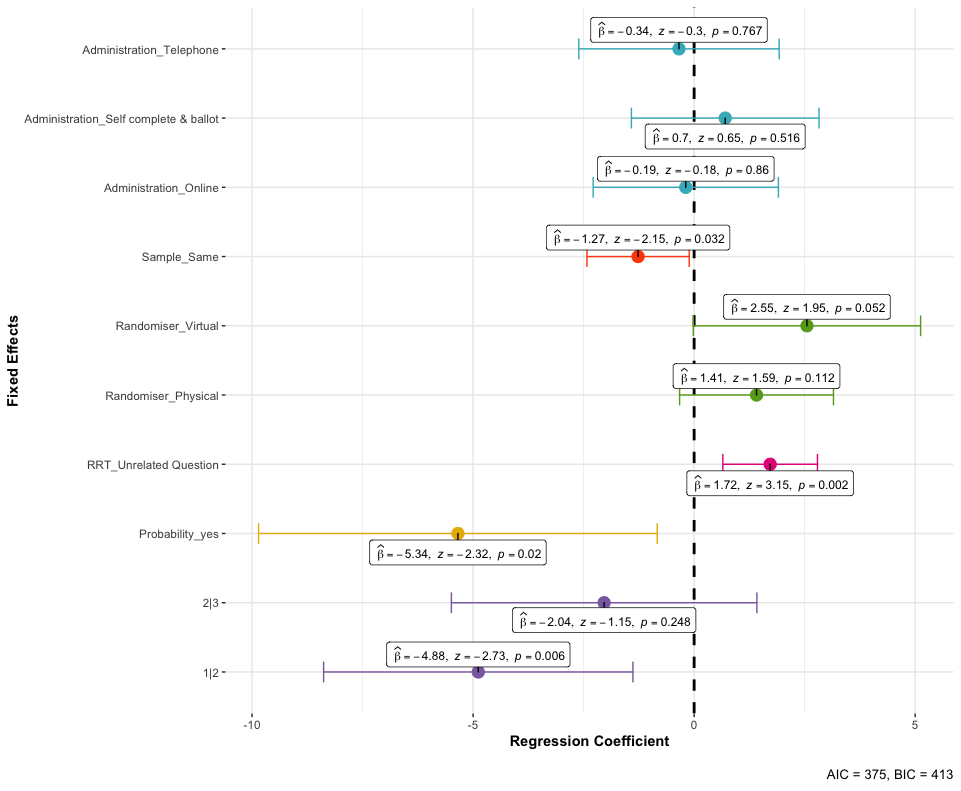


Figure B3. Box & whisker plot showing regression coefficients with 95% CIs from an ordered-logistic regression with random-effect for study, fitted to assess which factors influence whether RRTs provide higher, lower or non-significant prevalence estimates, compared to direct questions. Comparisons were made between 231 questions across 32 studies. Incept/reference levels were: Forced-response RRT design, Personal number randomising device (e.g. birth date or month), DQ & RRT responses collected from separate samples, face-to-face administration mode; 1= RRT performed worse than DQ, 2 = No significant difference, 3= RRT performed better than DQ

Table B2. Results from six validation studies included in the review. Level of validation data refers to whether validation data was collected at the individual respondent level, or the group level. In the Estimated Prevalence columns, values in *()* show the % difference between the known prevalence and the estimated RRT prevalence.

| **Study** | **Topic** | **RRT Method** | **Validation data** | **Level of validation data** | **Known prevalence** | **Estimated prevalence** *(difference)* | | |
| --- | --- | --- | --- | --- | --- | --- | --- | --- |
|  |  |  |  |  |  | **RRT** | **DQ** | **UCT** |
| Rosenfeld et al. 2016 | Voting against anti-abortion bill in 2011 Mississippi elections | Forced-response | Voting records | Individual | 65.3% | 59.4%  (-5.9%) | 30%  (-35.3%) | 56.2%  (-9.1%) |
| Höglinger & Jann, 2018 | Cheating in an experimental prediction game | Unrelated-Question | Known probability of number selection | Individual | 26.13% | 3.74%  (-22.4%) | - | - |
|  |  | Forced-response |  | Individual | 26.53% | 0.85%  (-25.7%) | - | - |
|  | Cheating in an experimental dice game | Unrelated-Question | Observation (Dice roll recorded) | Individual | 5.01% | 5.23%  (+0.2%) | - | - |
|  |  | Forced-response |  | Individual | 5.20% | –1.94%  (-7.1%) | - | - |
| Kirchner, 2015 | Receipt of basic income support | Forced-response | Government records | Group | 100% | 85.4%  (-14.6%) | 90.6%  (-9.4%) | -  - |
| Wolter & Preisendorfer 2013 | Committing minor offences | Forced-response | Court records | Individual | 100% | 59.6%  (-40.4%) | 57.5  (-42.5%) | - |
| Bova et al. 2018 | Breaching of recreational angling regulations | Forced-response | Covert observation | Individual | 100% | 44.3%  (-55.7%) | 46.5%  (-53.5%) | - |

Table B3. Studies identified in the review that used a statistical model to quantify the prevalence of self-protective responses.

UQ-RRT = unrelated-question RRT design, FR-RRT = forced-response RRT design, CDM = Cheating Detection Model

| **ID** | **Study** | **Research Question** | **RRT design** | **Post-hoc analyses** | **Sample size**  **(n)** | **Estimates (%)** | | |
| --- | --- | --- | --- | --- | --- | --- | --- | --- |
|  |  |  |  |  |  | **Honest yes** | **Honest no** | **Cheaters** |
| S1 | Elbe et al. 2018 | Have you intentionally used forbidden substances and/or forbidden methods in order to enhance your sporting performance in competitions during the last season? | UQ-RRT | Total cheating detection model^2^ | 624 | 0 | 69.4 | 30.6 |
|  |  | Have you ever intentionally used forbidden substances and/or forbidden methods in order to enhance your sporting performance in competitions?” |  |  | 581 | 3.1 | 74 | 22.9 |
| S2 | Frenger et al. 2016 | Doping last year | UQ-RRT | CDM^2^ | 616 | 4.1 | 70.2 | 25.8 |
|  |  | Doping ever |  |  | 786 | 3.6 | 88.4 | 8 |
|  |  | Self-medication last year |  |  | 616 | 21.4 | 78.6 | 0 |
|  |  | Self-medication ever |  |  | 786 | 49.2 | 27.9 | 22.9 |
| S3 | Moshagen & Musch, 2012 | Political asylum | FR-RRT | Multiple-Issues cheating detection model^3^ | 1053 | 31 | 49.6 | 19.4 |
|  |  | Homosexuality |  |  | 1053 | 49.6 | 30.4 | 20 |
|  |  | Renewable energy |  |  | 1053 | 49.2 | 44.8 | 6 |
| S4 | Pitsch et al. 2011 | Have you ever used illicit drugs or methods in order to enhance your sporting performance? | FR-RRT | CDM^1^ | 1556 | 10.2 | 65.2 | 24.7 |
|  |  | Have you used illicit drugs or methods in order to enhance your sporting performance in the current season? |  |  | 1556 | 9.6 | 65 | 25.4 |
| S5 | Moshagen et al. 2011 | Cooperation in an experimental game | FR-RRT | CDM^1^ | 1361 | 50.1 | 41.3 | 8.6 |
| S6 | Ostapczuk et al. 2011 | Have you ever intentionally and for a considerable time not taken medication prescribed by a physician as directed? | FR-RRT | CDM^1^ | 473 | 32.7 | 20.2 | 47.1 |
| S7 | Ostapczuk et al. 2008 | Assuming that you have a 20-year-old daughter. Would you mind her having a relationship with a dark-skinned Nigerian (high education) | FR-RRT | CDM^1^ | 217 | 30.1 | 52.7 | 17.2 |
|  |  | Assuming that you have a 20-year-old daughter. Would you mind her having a relationship with a dark-skinned Nigerian (low education) |  |  | 259 | 38.1 | 23.8 | 38.1 |
| S8 | Frenger et al. 2019 | Have you ever been approached to participate in a competition manipulation or match fixing? | UQ-RRT | No cheater detection model^2^ | Unclear | 8.4 | 89.6 | 2 |
|  |  | Have you ever been personally involved in a competition manipulation or match fixing? |  |  | Unclear | 7.5 | 82.6 | 10 |
|  |  | Have you ever observed an active effort to influence a referee or official with the objective of competition rigging? |  |  | 351 | 33 | 61.6 | 5.4 |
|  |  | Have you ever personally exerted an influence on a referee with the objective to manipulate a competition? |  |  | 351 | 4.9 | 90.5 | 4.6 |
| S9 | Schroter et al. 2016 | Have you taken substances to increase your physical performance in the past 12 months that are only available at a pharmacy, at the doctor’s office or on the black market? (Frankfurt) | FR-RRT | CDM^1^ | 1001 | 11.9 | 23.2 | 64.9 |
|  |  | Have you taken substances to increase your mental performance in the past 12 months that are only available at a pharmacy, at the doctor’s office or on the black market? (Frankfurt) |  |  | 1001 | 9.4 | 31.5 | 59.1 |
|  |  | Have you taken substances to increase your physical performance in the past 12 months that are only available at a pharmacy, at the doctor’s office or on the black market? (Wiesbaden) |  |  | 482 | 4.2 | 41.9 | 53.9 |
|  |  | Have you taken substances to increase your mental performance in the past 12 months that are only available at a pharmacy, at the doctor’s office or on the black market? (Wiesbaden) |  |  | 482 | 0.7 | 54.8 | 44.5 |

^1^ following design of Clark & Desharnais, 1998 referenced, ^2^ following design of Feth et al. 2017, ^3^ As proposed by author

Figure B4. Probability tree and example of the Cheating Detection Model developed by Clark & Desharnais (1998) where *p* represents the probability of the respondent being asked to answer the sensitive question.
